# Molecular Characterization and Promoter Activity Analysis of the Porcine Convergent Gene Pair *ABCF1* and *PPP1R10*

**DOI:** 10.3390/ani16213416

**Published:** 2026-10-29

**Authors:** Tian Wang, Yuanchen Mei, Qiang Yang

**Affiliations:** National Key Laboratory for Pig Genetic Improvement and Germplasm Innovation, Ministry of Science and Technology, Jiangxi Agricultural University, Nanchang 330045, China; wangtian@stu.jxau.edu.cn (T.W.); meiyuanchen@stu.jxau.edu.cn (Y.M.)

**Keywords:** pig, *ABCF1* and *PPP1R10*, molecular characterization, core promoter, genomic safe harbor

## Abstract

The intergenic regions of porcine convergent gene pairs frequently maintain an open chromatin configuration with a permissive transcriptional microenvironment, rendering them promising candidates for genomic safe harbors (GSHs). However, the molecular features and promoter activities of the convergent gene pair *ABCF1* and *PPP1R10* remain uncharacterized, impeding the utilization of their potential as GSHs. To address this, we systematically predicted cis-regulatory elements (including CpG islands) of the two genes in silico, evaluated the transcriptional activities of serially truncated promoter fragments by dual-luciferase reporter assays in porcine fetal fibroblasts (PFFs) and PK-15 cells, compared their promoter regions across seven species, and characterized the protein architectures and flanking genomic landscapes. The ABCF1 promoter contained canonical TATA, CCAAT, and GC boxes alongside three CpG islands, and its shortest fragment, P217 (−217 to −1 bp), exhibited the highest activity in both cell lines. The PPP1R10 promoter possessed a triple TATA-box arrangement without detectable CpG islands, and its core fragment P211 (−211 to −1 bp) exhibited the highest activity among all truncated fragments in both cell lines and showed numerically higher activity than the simian virus 40 (SV40) promoter in PK-15 cells. No canonical oncogenes were identified within 100 kb upstream or downstream of the intergenic region between the two genes. Both ABCF1 and PPP1R10 are intracellular soluble proteins lacking transmembrane domains. ABCF1 is predominantly composed of α-helices, localizes in the cytoplasm and at the nuclear membrane, and modulates cytoplasmic translation. By contrast, PPP1R10 is enriched in random coils, specifically associates with nuclear chromatin, and participates in the PP1 phosphatase regulatory network. Taken together, our study delineates the core active promoter regions of both genes and highlights the potential of this chromosomal interval as a candidate genomic safe-harbor locus, a hypothesis awaiting further experimental validation.

## Simple Summary

Intergenic intervals between convergent gene pairs in pigs often contain accessible chromatin, making them suitable for genomic safe harbor screening. However, the *ABCF1*-*PPP1R10* locus remains poorly understood regarding its promoter function. Here, we mapped its cis-regulatory sequences, tested serially truncated promoter constructs, and analyzed the protein structures and surrounding genomic context. We successfully delineated the minimal functional promoter segments, identified divergent promoter motifs and protein features, and found no evidence of nearby oncogenes. Overall, these findings provide fundamental evidence to support this locus as a novel genomic safe-harbor candidate. Nevertheless, further functional validation is required before this locus can be used to generate transgenic pig models of human diseases.

## 1. Introduction

Convergent gene pairs, whose two protein-coding genes are transcribed from opposite DNA strands toward a shared intergenic interval, constitute a widespread genomic architecture in eukaryotes [1]. Genome-wide epigenomic profiling demonstrates that intergenic regions frequently maintain permissive histone marks such as H3K27ac and H3K4me3 across diverse cell types [2,3]. Such transcriptionally permissive epigenetic landscapes theoretically meet core criteria for genomic safe harbor (GSH) loci: accessible chromatin supports sustained transgene expression, while appropriate spacing between endogenous genes minimizes disruption to native transcriptional networks [4]. Genomic safe harbors are defined as discrete chromosomal regions enabling stable transgene integration without perturbing host gene function, inducing oncogenic transformation, or triggering progressive transgene silencing [5,6]. Precise site-specific transgene insertion into validated GSH sites is indispensable for generating genetically modified pigs applied in agricultural genetic improvement, human disease modeling, and xenotransplantation [7,8,9].

Despite its critical value, the repertoire of functionally verified porcine GSH candidates remains limited. To date, reported candidates include the *ACTB* [10] and *GAPDH* loci [11], the H11 safe-harbor locus [12], and the porcine AAVS1-homologous region [13]. Nevertheless, these existing sites exhibit notable drawbacks: intragenic integration may perturb endogenous gene function, and multiple purported safe loci display gradual transgene silencing and expression heterogeneity during long-term cell passaging [4]. Similar clonal variability in reporter expression has been documented at the human *AAVS1* locus in induced pluripotent stem cells, compromising experimental reproducibility [14]. Accordingly, identification and functional validation of additional high-quality porcine GSH candidates are urgently required.

Over the past decade, immune-related quantitative trait loci (QTL) affecting blood-cell parameters and T lymphocyte subpopulations have been repeatedly identified in independent pig populations [15,16,17], with several of these signals mapped to porcine chromosome 7 [16,17]. Successive genome-wide association studies (GWASs) have repeatedly implicated this chromosome, with *ABCF1* and *PPP1R10* identified as plausible positional candidate genes for immune-related traits. *ABCF1* encodes a cytoplasmic ATP-binding cassette protein lacking transmembrane domains; it acts as a translational initiation regulator and serves as an important modulator of innate immunity, inflammatory responses, and antiviral defense [18,19]. *PPP1R10* produces PNUTS, a core regulatory subunit of the PTW/PP1 phosphatase complex governing RNA polymerase II pause release, transcription elongation, and MYC protein stability [20,21,22,23]. Strikingly, *ABCF1* and *PPP1R10* form a typical convergent gene pair separated by an approximately 6.5 kb shared intergenic region (chr7:23,143,436–23,149,933, between the curated RefSeq transcripts) on porcine chromosome 7 [24]. Although robust genetic associations exist between this gene pair and porcine immune phenotypes, the promoter architecture and transcriptional regulatory characteristics of porcine *ABCF1* and *PPP1R10* remain largely uncharacterized. Moreover, no study has evaluated whether the genomic interval surrounding this convergent gene pair can serve as a novel porcine GSH candidate.

Here, we systematically characterized the 5′-flanking sequences of porcine *ABCF1* and *PPP1R10* through in silico promoter prediction, dual-luciferase reporter assays across different cell types, and comparative structural modeling of the encoded proteins. We also assessed the genomic context within a 200-kb window (±100 kb around the midpoint of the intergenic region) surrounding both promoters to evaluate biosafety for exogenous gene integration. Our results identify the core promoter regions and confirm their capacity to drive robust transcription, with the *PPP1R10* minimal promoter exhibiting a distinctive triple TATA-box architecture and activity numerically exceeding that of the SV40 promoter in PK-15 cells. Comparative genomic analysis supports the potential of this chromosomal interval as a novel porcine safe harbor candidate locus, though additional functional validation is required. This work provides a foundational framework for subsequent CRISPR/Cas9-mediated site-specific transgene integration and long-term expression stability validation in pigs. The objective of the present study was therefore to characterize the core promoter regions and genomic context of the porcine convergent gene pair *ABCF1*/*PPP1R10* and to evaluate the potential of their intergenic interval as a candidate genomic safe harbor.

## 2. Materials and Methods

### 2.1. Bioinformatics Analysis of the 5′-Flanking Regions of Porcine ABCF1 and PPP1R10

All genomic coordinates in this study refer to the pig reference assembly Sscrofa11.1 (GCF_000003025.6; NC_010449.5; NCBI annotation release 106). The *PPP1R10* promoter region was defined relative to the coding-sequence (CDS) start of the curated RefSeq transcript NM_001123165.1 (chr7:23,158,750 on the minus strand); the annotated *PPP1R10* gene span extends to chr7:23,166,933 because the predicted isoform XM_013996564.2 uses an alternative upstream first exon located approximately 8.2 kb distal to the ATG start codon. The 3000 bp 5′-flanking upstream sequences of porcine *ABCF1* (NC_010449.5:23123338-23126337) and *PPP1R10* (NC_010449.5:c23161750-23158751) were retrieved from the NCBI database. Core promoter regions were predicted using the Neural Network Promoter Prediction (NNPP) tool (version 2.2) [25]. CpG islands were identified by MethPrimer [26] with unified criteria: sequence length > 100 bp, GC content > 50%, and observed/expected CpG ratio > 0.6. Putative transcription factor binding sites were screened using FIMO from the MEME-Suite [27] against the JASPAR 2026 CORE vertebrate non-redundant motif database (*p* < 1 × 10^−4^, scanning both DNA strands) [28], and the candidate sites were compared with experimental regulatory evidence from the KnockTF 2.0 database [29]; for each transcription factor, only the binding site with the lowest *p*-value was retained, yielding 14 and 18 putative sites for *ABCF1* and *PPP1R10*, respectively. For cross-species conservation analysis, the 500 bp sequences immediately upstream of each start codon from human, mouse, rat, cattle, sheep, and zebrafish were aligned with MUSCLE [30], and mean pairwise nucleotide identities were calculated in DNAMAN 6.0 (Lynnon Biosoft, San Ramon, CA, USA); alignment views were rendered with ESPript 3.2 [31]. Detailed information on all bioinformatics software and databases is summarized in Table S1.

### 2.2. Construction of Truncated Promoter Luciferase Reporter Vectors

Based on bioinformatic prediction results, six serially truncated fragments of the *ABCF1* promoter (P217: −217 to −1 bp, P427: −427 to −1 bp, P905: −905 to −1 bp, P1241: −1241 to −1 bp, P2119: −2119 to −1 bp, P2997: −2997 to −1 bp) and five truncated fragments of the *PPP1R10* promoter (P211: −211 to −1 bp, P687: −687 to −1 bp, P1727: −1727 to −1 bp, P1939: −1939 to −1 bp, P2159: −2159 to −1 bp) were designed. The adenine of the ATG translation initiation codon was defined as the +1 site, with all upstream sequences numbered negatively. All promoter fragments were chemically synthesized (Tsingke Biotechnology, Beijing, China) and subcloned into the pGL3-Basic luciferase vector. A pGL3-Basic derivative carrying the SV40 promoter (pGL3-SV40; Tsingke Biotechnology, Beijing, China) was used as a positive control, and the empty pGL3-Basic vector served as a negative control. All recombinant plasmids were validated by Sanger sequencing prior to cell transfection. The pRL-TK vector expressing Renilla luciferase was co-transfected as an internal control to normalize transfection efficiency.

### 2.3. Cell Culture and Transfection

Porcine fetal fibroblasts (PFFs) were isolated from Large White pig fetuses and cultured in complete Dulbecco’s Modified Eagle’s Medium (DMEM; Gibco, Grand Island, NY, USA) supplemented with 12% fetal bovine serum (FBS; Gibco, Grand Island, NY, USA) and 1% penicillin–streptomycin (Gibco, Grand Island, NY, USA) mixture. The porcine kidney epithelial cell line PK-15 was obtained from the National Cell Bank of the Chinese Academy of Sciences (Shanghai, China) and cultured in the same complete medium. PFFs were used at passage 3 and PK-15 cells at passage 7 to obtain sufficient cell numbers for transfection while maintaining normal cell morphology and physiological status. Primary PFFs tend to show reduced proliferation and morphological alterations at later passages; passage 7 falls within the practical working range for this established PK-15 epithelial line after thawing. All cells were cultured at 37 °C in a humidified incubator with 5% CO_2_ and routinely verified to be mycoplasma-negative.

Two days before transfection, cells were seeded into 10 cm culture dishes and harvested for transfection upon reaching 80–90% confluence. For each reaction, 2 × 10^5^ cells were co-transfected with 2 μg of pGL3-series firefly luciferase plasmids and 100 ng of pRL-TK Renilla luciferase plasmid using the Lonza 4D-Nucleofector™ Core Unit (Lonza, Basel, Switzerland); the CA-137 program was applied twice per reaction for both cell types. Transfected cells were seeded into 6-well plates, and the culture medium was refreshed 6 h post-transfection.

### 2.4. Dual-Luciferase Reporter Assay

At 18 h after transfection, cells were harvested and fully lysed. Aliquots (50 μL) of the clarified lysate were dispensed into a white 96-well luminescence microplate. Firefly and Renilla luciferase activities were sequentially detected using a TECAN Infinite M200 PRO microplate reader (Männedorf, Switzerland) after the addition of the corresponding reaction substrates. For each well, the firefly value was divided by its paired Renilla value; these ratios served as the relative activity throughout, correcting for variation in transfection efficiency. Each recombinant plasmid was tested in three to five independent transfection batches per cell line (three for *ABCF1* in PFFs, four for *ABCF1* in PK-15 cells, five for *PPP1R10* in PFFs, and four for *PPP1R10* in PK-15 cells; 16 batches in total).

### 2.5. Structural and Functional Bioinformatics Analysis of Porcine ABCF1 and PPP1R10 Proteins

Amino acid sequences of *ABCF1* and *PPP1R10* from seven species (pig, human, mouse, rat, cattle, sheep, and zebrafish) were retrieved from the UniProt database for cross-species conservation analysis. Protein sequences were aligned with MUSCLE as implemented in MEGA 11 (v11.0.13) [32], mean pairwise amino-acid identities were derived from p-distance matrices, and alignment views were rendered with ESPript 3.2 [31]. Protein physicochemical properties were analyzed using ProtParam [33], while hydrophilicity and hydrophobicity profiles were predicted via ProtScale based on the Kyte-Doolittle algorithm [34]. SignalP 5.0 [35] and TMHMM 2.0 [36] were used to predict signal peptides and transmembrane domains, respectively. Protein secondary structures were analyzed using GOR4 [37], and conserved functional domains were identified via the InterPro database [38]. Tertiary-structure homology modelling was performed using SWISS-MODEL [39]. For ABCF1, the experimentally solved PDB template 5zxd.2.A was used (99.26% sequence identity within the modelled region). For porcine PPP1R10 (UniProt: Q767K9), for which no full-length experimentally solved structure is available, a homology model was generated using SWISS-MODEL with the AlphaFold DB entry AF-Q767K9-F1 as template; the corresponding per-residue pLDDT and Predicted Aligned Error (PAE) values were obtained directly from AlphaFold DB for confidence assessment. All structural visualizations in this study were rendered using the SWISS-MODEL viewer. The STRING database (v12.0) [40] with a confidence threshold of 0.700 was used to construct protein–protein interaction networks of porcine ABCF1 and PPP1R10. Detailed information on all analytical tools is listed in Table S1.

### 2.6. Statistical Analysis

All dual-luciferase assay data were normalized within each experimental batch, with the mean activity of the pGL3-SV40 positive control set to 10 for unified scaling. Each construct was evaluated in the same independent transfection batches described in Section 2.4 (16 batches in total across the two cell lines and two genes); technical replicate wells (two to four per batch) were averaged within each batch, and the resulting batch means were analyzed by a randomized-block ANOVA with batch as a blocking factor to remove batch-to-batch transfection effects, followed by Tukey’s HSD test. To compare each construct with the positive control, batch means were tested against the normalized control value of 10 by one-sample *t* tests with Holm correction within each panel. The pGL3-SV40 positive control served as an external reference for normalization and as a control for transfection efficiency rather than as a treatment group, and the pGL3-Basic negative control reflected near-background promoter activity; these two controls were therefore excluded from the main statistical model and are shown for reference only. Statistical significance was defined as *p* < 0.05. Results are presented as box plots (interquartile range and median) with individual technical replicate wells; group comparisons are shown with lowercase letters, and constructs sharing a letter do not differ significantly (*p* > 0.05). Data visualization and statistical analyses were performed using R (v4.5.2) [41] with the ggplot2 package (v4.0.2) [42]. Within each batch, the intra-batch coefficient of variation (CV) of the SV40 technical-replicate wells (median 3.4%, range 0.05–10.3% across 16 batches with ≥2 replicates) reflected typical transfection-assay variation, which was substantially smaller than the between-construct differences tested in the main analysis.

## 3. Results

### 3.1. Bioinformatic Characterization of the 5′-Flanking Regulatory Regions of Porcine ABCF1 and PPP1R10

**Figure 1:**
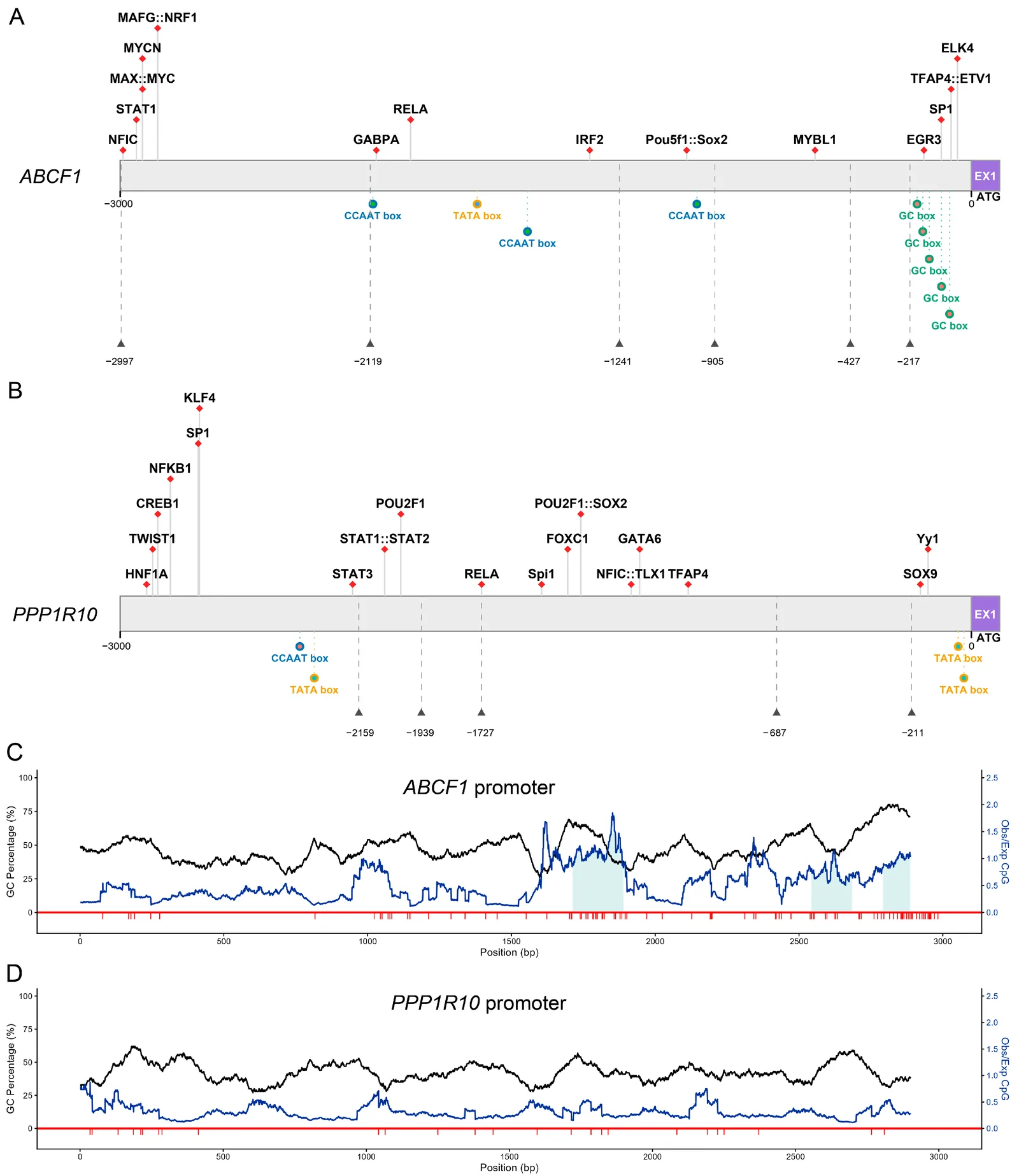
In silico prediction of cis-regulatory elements and CpG islands in the 5′-flanking regions of porcine *ABCF1* (**A**,**C**) and *PPP1R10* (**B**,**D**). (**A**,**B**) Predicted transcription-factor binding sites (red diamonds; names in Tables S2 and S3) and cis-acting elements (circles: CCAAT, blue; TATA, orange; GC, green); gray triangles, boundaries of serially truncated fragments; EX1, first exon; ATG, translation-initiation adenine (defined as position + 1). (**C**,**D**) CpG-island prediction: light-blue shading, islands (three in *ABCF1*, none in *PPP1R10*); black curve, GC percentage; blue curve, observed/expected CpG ratio; red line, island threshold. The x-axis indicates the base-pair position relative to the ATG start codon.

To identify the core promoter regions of porcine *ABCF1* and *PPP1R10*, the 5′-flanking sequences of both genes were systematically dissected. The *ABCF1* 5′-flanking region displayed a canonical eukaryotic promoter architecture, containing nine valid cis-acting elements (1 TATA, 3 CCAAT, and 5 GC boxes) and three CpG islands (Figure 1A,C). In addition, 14 high-confidence TF-binding sites were predicted, involving MAFG::NRF1, SP1, TFAP4::ETV1, ELK4, MYCN, MAX::MYC, RELA, EGR3, Pou5f1::Sox2, MYBL1, GABPA, NFIC, STAT1, and IRF2 (Figure 1A and Table S2). Among these, MAFG::NRF1 and SP1 exhibited the most significant predicted binding, suggesting potentially pivotal roles in *ABCF1* transcription; notably, the SP1 site lies within the minimal P217 fragment. Five high-confidence core promoter candidates were further identified, all with prediction scores ≥ 0.85 (Table 1).

The *PPP1R10* 5′-flanking region contained four valid cis-acting elements (three TATA boxes and one CCAAT box), featuring a proximal tandem TATA pair, but no CpG islands meeting the established criteria (Figure 1B,D). A total of 18 high-confidence TF-binding sites were predicted (Figure 1B and Table S3). Spi1 and FOXC1 exhibited the most significant predicted binding, indicating potentially prominent roles in *PPP1R10* transcription. Three high-confidence core promoter candidates were identified, all with scores ≥ 0.85 (Table 1). The experimentally active core promoters were subsequently defined by the serial-truncation reporter analysis (Section 3.2), in which the shortest fragments P217 and P211 conferred the highest activity.

We further evaluated the genomic context flanking the *ABCF1*-*PPP1R10* intergenic region to assess its suitability as a genomic safe-harbor candidate. Analysis of the genomic context within the ±100 kb window centered on the midpoint of the intergenic region (chr7:23,046,684–23,246,684) identified 13 protein-coding genes (Figure S1A,B), none of which is annotated as a known oncogene in the COSMIC Cancer Gene Census. Public pig epigenome datasets further show that the intergenic interval carries a discrete regulatory element co-marked by ATAC, H3K27ac and H3K4me3 located 0.6–1.1 kb from the candidate insertion site, whereas the site itself is free of marked regulatory peaks (Figure S1C). This satisfies the fundamental sequence-level criteria for a genomic safe-harbor candidate locus. In addition, comparison of the 500 bp sequences upstream of the initiation codon of *ABCF1* and *PPP1R10* across seven species (pig, human, mouse, rat, cattle, sheep, and zebrafish) revealed moderate to relatively high conservation, with mean identities of 60.39% and 60.27%, respectively (Figure S2).

### 3.2. Transcriptional Activity of Serially Truncated Promoter Fragments

**Figure 2:**
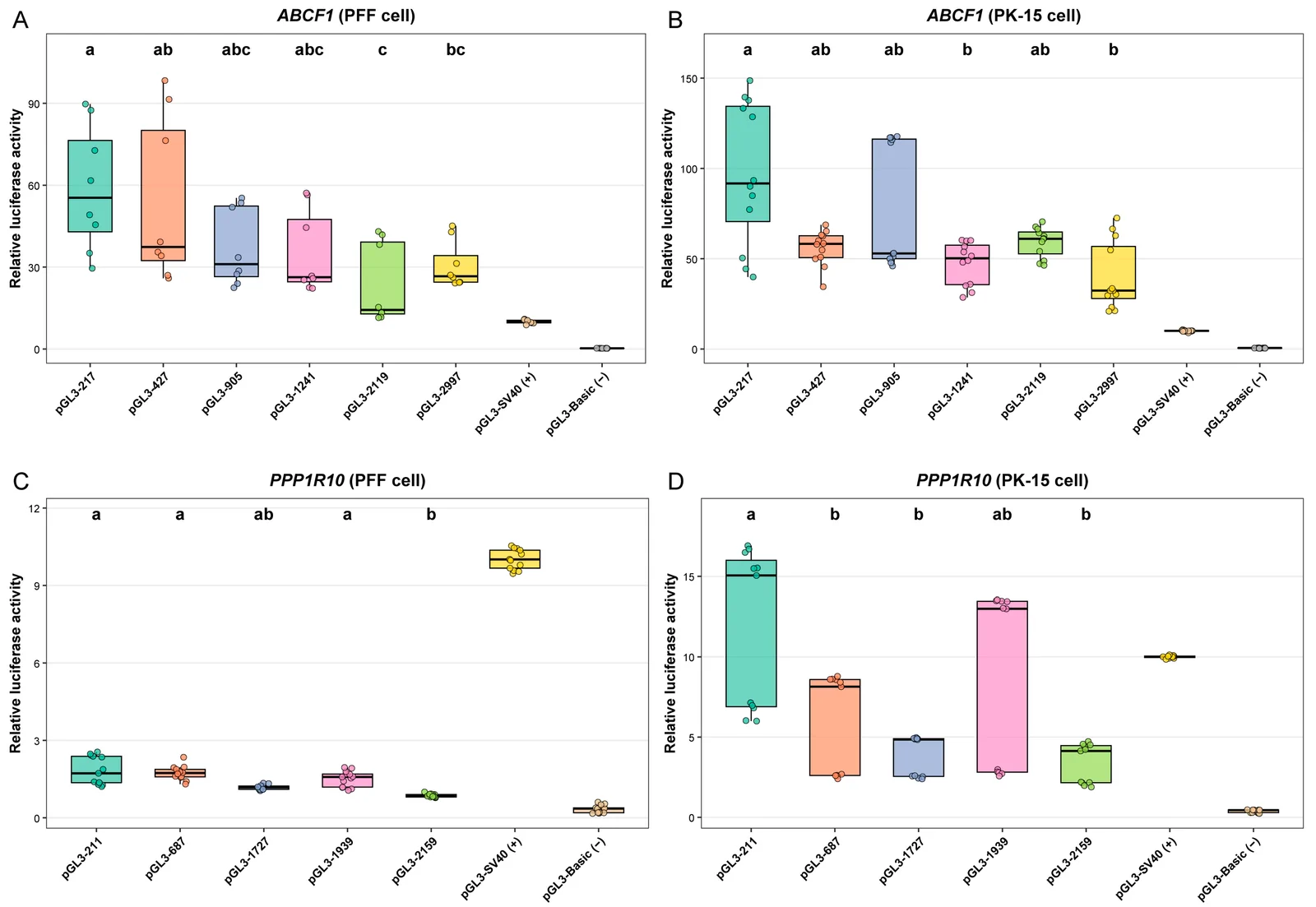
Dual-luciferase reporter assay of serially truncated ABCF1 and PPP1R10 promoter constructs. (**A**) ABCF1 in PFF cells; (**B**) ABCF1 in PK-15 cells; (**C**) PPP1R10 in PFF cells; (**D**) PPP1R10 in PK-15 cells. Activity was normalized to the mean pGL3-SV40 activity per batch (set to 10). Boxplots show median and interquartile range; points show individual technical replicate wells; statistical comparisons were performed on batch means (2–4 technical replicates averaged per independent transfection batch) rather than on individual wells. Different lowercase letters denote significant differences among constructs (randomized-block ANOVA with batch as blocking factor, followed by Tukey’s HSD; *p* < 0.05); shared letters, no significant difference. pGL3-SV40 and pGL3-Basic controls were excluded from the model and are shown for reference only. The y-axis represents relative luciferase activity (firefly-to-Renilla ratio).

Dual-luciferase reporter assays in porcine fetal fibroblasts (PFFs) and PK-15 cells verified the transcriptional activity of all serially truncated *ABCF1* and *PPP1R10* promoter fragments (Figure 2). The empty pGL3-Basic vector displayed extremely low background activity, while the pGL3-SV40 positive control exhibited stable normalized activity (mean = 10), validating the reliability of the experimental system.

For ABCF1 (Figure 2A,B), all six truncated fragments exhibited significantly higher activity than the pGL3-SV40 positive control in both cell lines (*p* < 0.05). The shortest fragment, P217 (−217 to −1 bp), displayed the highest activity among all truncations in both PFFs and PK-15 cells. As fragment length increased, transcriptional activity gradually declined, with a modest increase observed at P2997 (−2997 to −1 bp) in PFFs, and at P2119 (−2119 to −1 bp) in PK-15 cells before declining again. In PFFs, P427 (−427 to −1 bp) also showed higher activity than the distal longer fragments, most notably the lowest-activity fragment P2119 (−2119 to −1 bp; *p* < 0.05), whereas in PK-15 cells, P217 (−217 to −1 bp) remained the sole fragment with maximal activity.

For PPP1R10 (Figure 2C,D), the five truncated fragments differed significantly in activity, with a consistent trend across both cell lines. The shortest fragment, P211 (−211 to −1 bp), exhibited the highest activity among all truncations. As fragment length increased, transcriptional activity gradually declined, with a moderate increase at P1939 (−1939 to −1 bp) before declining again. Notably, P211 (−211 to −1 bp) in PK-15 cells exhibited numerically higher activity than the pGL3-SV40 positive control.

These results indicate that the core functional promoters of both genes reside within the shortest fragments tested, approximately 217 bp for *ABCF1* and 211 bp for *PPP1R10*, both located upstream of the translation initiation site.

### 3.3. Physicochemical Properties and Subcellular Localization of Porcine ABCF1 and PPP1R10

Having characterized the promoter activities of the two genes, we next examined the biochemical properties and subcellular localization of the encoded proteins. Full-length amino acid sequences of porcine ABCF1 (UniProt K7GL02, TrEMBL, 824 aa) and PPP1R10 (UniProt Q767K9, Swiss-Prot reviewed, 925 aa) were retrieved from UniProt. The two proteins exhibited markedly distinct biochemical profiles (Table 2 and Table S4). ABCF1 was an acidic protein (pI 5.94), whereas PPP1R10 was a basic protein (pI 9.25); both were hydrophilic, had instability indices above 40, and shared comparable half-life predictions.

**Figure 3:**
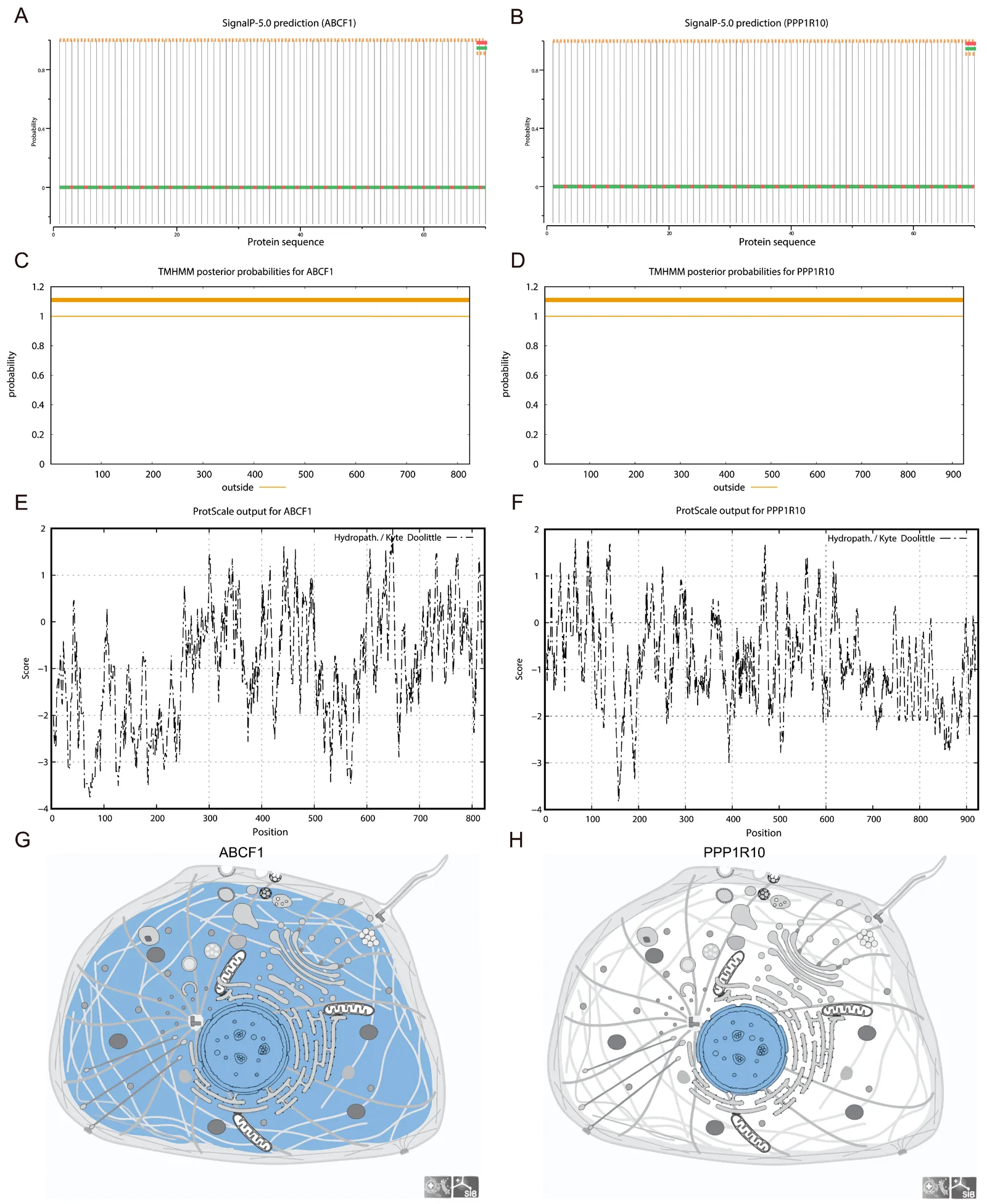
Bioinformatic prediction of signal peptide, transmembrane helices, hydrophobicity and subcellular localization of porcine ABCF1 (**A**,**C**,**E**,**G**) and PPP1R10 (**B**,**D**,**F**,**H**). (**A**,**B**) SignalP 5.0 signal-peptide prediction; (**C**,**D**) TMHMM 2.0 transmembrane-helix prediction; (**E**,**F**) Kyte-Doolittle hydrophilicity/hydrophobicity profiles (ProtScale); (**G**,**H**) predicted subcellular localization.

SignalP 5.0 predicted no classical secretory signal peptide for either protein (non-secretory probabilities approaching 1; Figure 3A,B). TMHMM 2.0 predicted no transmembrane helices for either protein (Figure 3C,D). ProtScale hydrophilicity profiles revealed no extensive continuous hydrophobic stretches (Figure 3E,F), consistent with the GRAVY values. UniProt Gene Ontology subcellular annotations indicated that ABCF1 was distributed in the cytoplasm, nuclear membrane, and nucleoplasm, whereas PPP1R10 was specifically localized to the nucleus, associating with chromatin and the PTW/PP1 phosphatase complex (Figure 3G,H). Together, these predictions indicate that both ABCF1 and PPP1R10 are intracellular soluble proteins devoid of signal peptides and transmembrane domains.

### 3.4. Secondary Structure, Conserved Domains, and Homology Modeling of Porcine ABCF1 and PPP1R10

**Figure 4:**
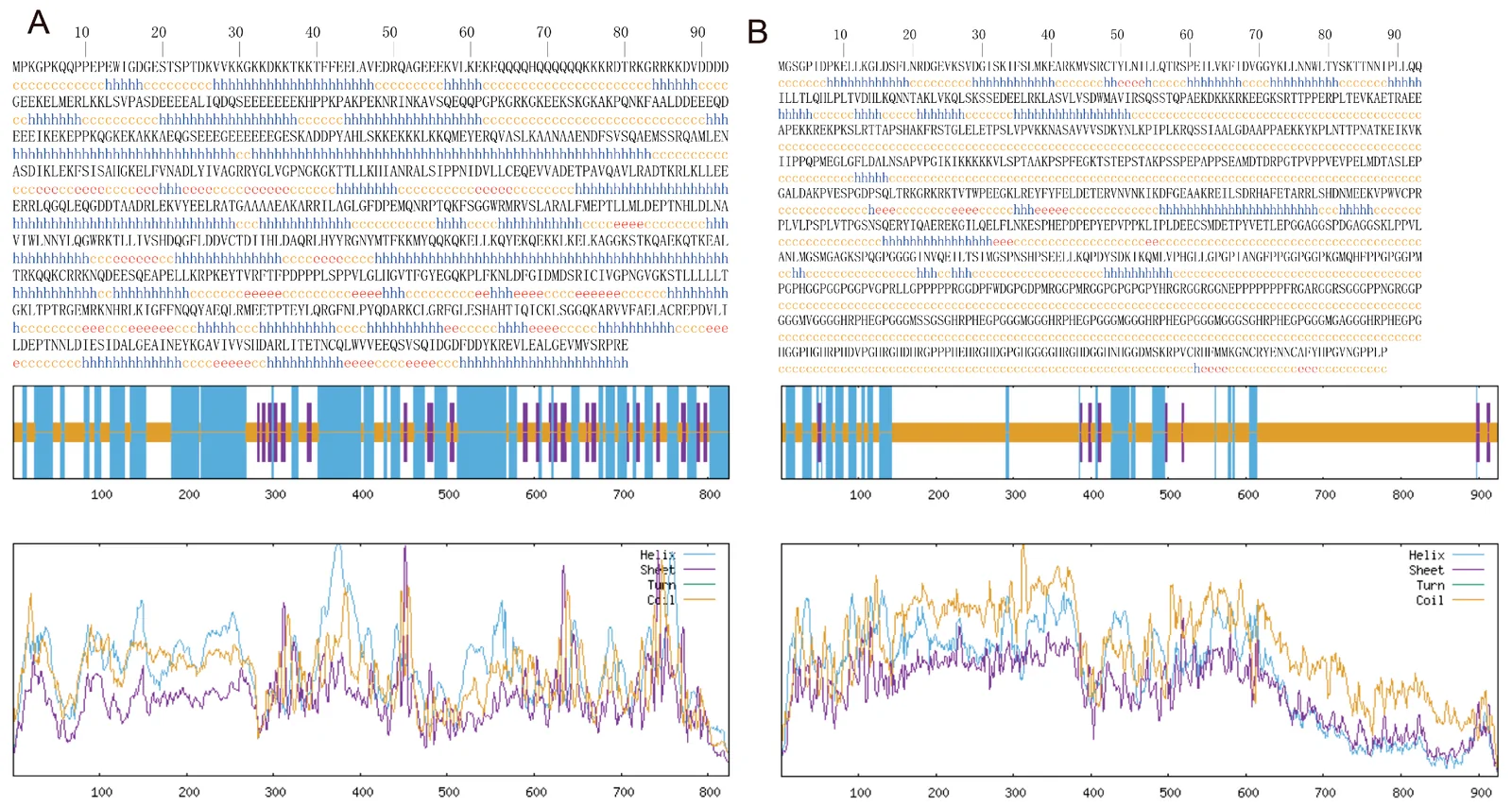
Secondary-structure prediction of porcine ABCF1 (**A**) and PPP1R10 (**B**) generated by GOR4. Top, amino acid sequences annotated with secondary-structure elements following the native GOR4 colour scheme: blue h, α-helix; red e, extended β-strand; orange c, random coil. Middle, block representation of hard-assigned secondary-structure residues, using the same colour definitions as the bottom-panel legend. Bottom, residue-wise probability curves, with embedded legend: helix (light blue), sheet (purple), turn (green), coil (orange). No β-turn residues were assigned by GOR4, so no turn curve appears in the bottom curves.

**Figure 5:**
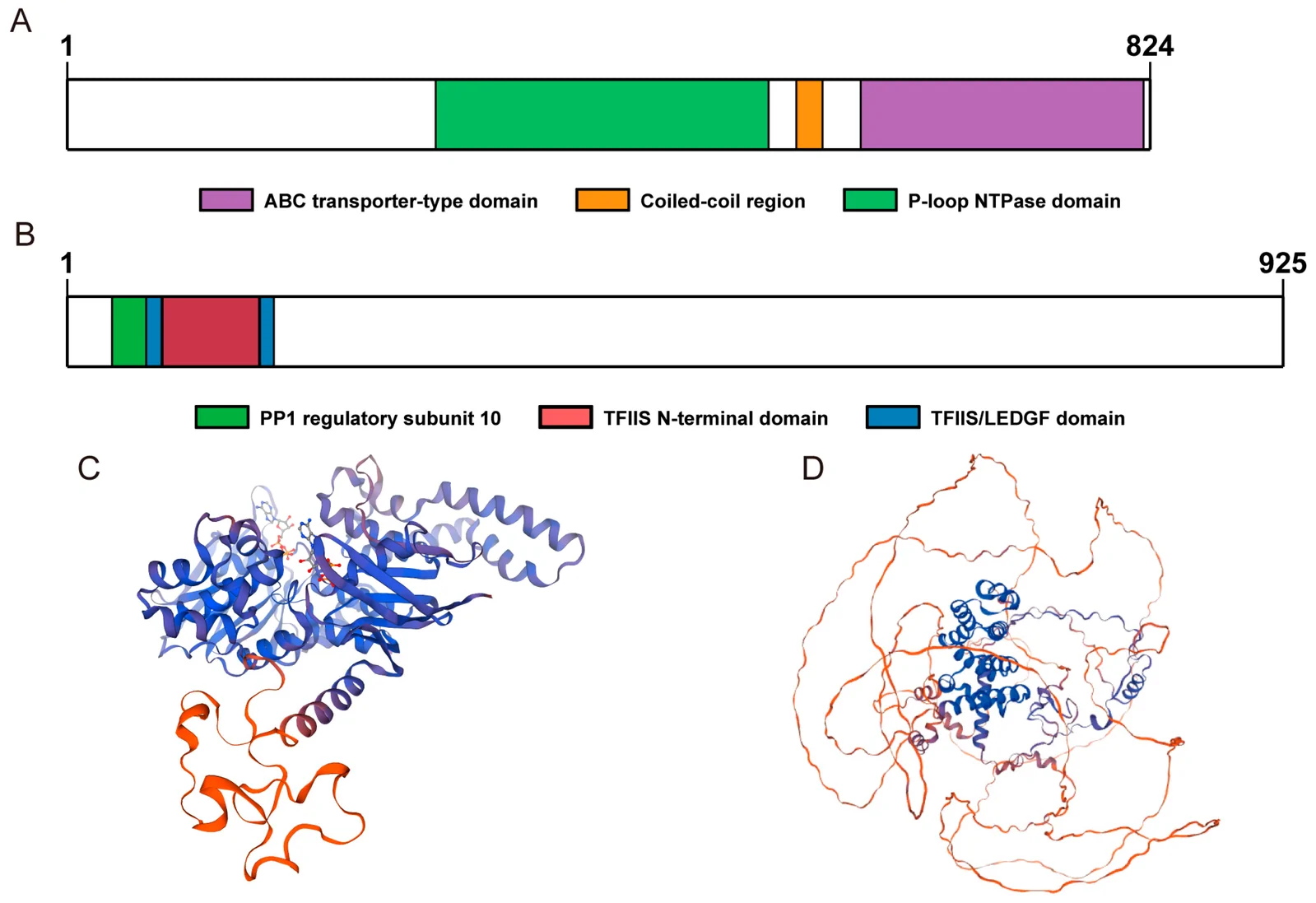
Conserved-domain annotation of porcine ABCF1 (**A**)-PPP1R10 (**B**); SWISS-MODEL homology model for ABCF1 (**C**) and SWISS-MODEL homology model for PPP1R10 (**D**). (**A**,**B**) Domain architecture, with the TFIIS N-terminal domain (red) drawn as a semi-transparent layer over the TFIIS/LEDGF domain (blue) in (B); (**C**,**D**) ribbon representation of the predicted tertiary structure. The ABCF1 model (**C**) was generated at moderate confidence (GMQE = 0.51; QMEANDisCo global score = 0.79 ± 0.05); the PPP1R10 model (**D**) was generated by SWISS-MODEL using AlphaFold DB entry AF-Q767K9-F1 as template; the per-residue pLDDT values were obtained from AlphaFold DB, yielding an average pLDDT of 57.81 (Low). In total, 28.6% of residues fall into high-confidence regions (15.1% very high, pLDDT > 90; 13.5% high, 70 < pLDDT < 90; 16.0% low, 50 < pLDDT < 70; 55.4% very low, pLDDT < 50). Both structural predictions should therefore be interpreted with caution. A Predicted Aligned Error (PAE) plot for this model is provided as Supplementary Figure S3, confirming that high-confidence prediction is limited to the folded N-terminal region. Model parameters are detailed in Section 3.4.

GOR4 predictions revealed distinct conformational architectures between the two proteins (Figure 4). ABCF1 was predominantly composed of α-helical (52.67%) and random coils (37.50%), with smaller proportions of extended strands (9.83%) (Figure 4A), whereas PPP1R10 was overwhelmingly composed of random coils (69.41%), with lower proportions of α-helices (22.49%), extended strands (8.11%) (Figure 4B). According to the GOR4 hard-assignment output, no β-turn residues were predicted for either protein (0.00%), hence no turn curve appears in Figure 4. InterPro multi-database scanning (CDD, Pfam, SMART, and Gene3D) identified the conserved functional domains shown in Figure 5. ABCF1 contained two core modules: a P-loop nucleoside triphosphate hydrolase domain (281–534 aa) and an ABC transporter signature domain (604–819 aa), with a coiled-coil interaction region (555–575 aa). Intrinsically disordered regions (MobiDB-Lite) were identified at 29–39, 209–221, and 538–581 aa. PPP1R10 contained an N-terminal PP1 regulatory subunit domain and a TFIIS N-terminal domain (73–147 aa) embedded within the TFIIS/LEDGF domain (35–158 aa), together with a C-terminal CCCH/C3H1-type zinc finger (891–919 aa) and disordered regions at 596–611, 679–693, and 846–871 aa.

SWISS-MODEL homology modelling generated the three-dimensional ribbon diagram of ABCF1 using template 5zxd.2.A [43], with 99.26% sequence identity in the modelled core region, a GMQE of 0.51, and a QMEANDisCo global score of 0.79 ± 0.05 (Figure 5C). The ribbon representation of PPP1R10 (Figure 5D) was generated by SWISS-MODEL using AlphaFold DB entry AF-Q767K9-F1 as a template and visualized with the SWISS-MODEL viewer (GMQE = 0.57; 100% identity; full-length coverage). For confidence assessment, per-residue pLDDT and PAE values were obtained directly from AlphaFold DB; no mechanistic inference is drawn from regions with pLDDT < 70, and structural interpretation is confined to the folded N-terminal region.

These results suggest that ABCF1 adopts a rigid α-helix-dominant conformation, whereas PPP1R10 adopts a flexible random-coil-dominant conformation.

### 3.5. Protein–Protein Interaction Networks, Functional Enrichment, and Cross-Species Conservation of Porcine ABCF1 and PPP1R10

**Figure 6:**
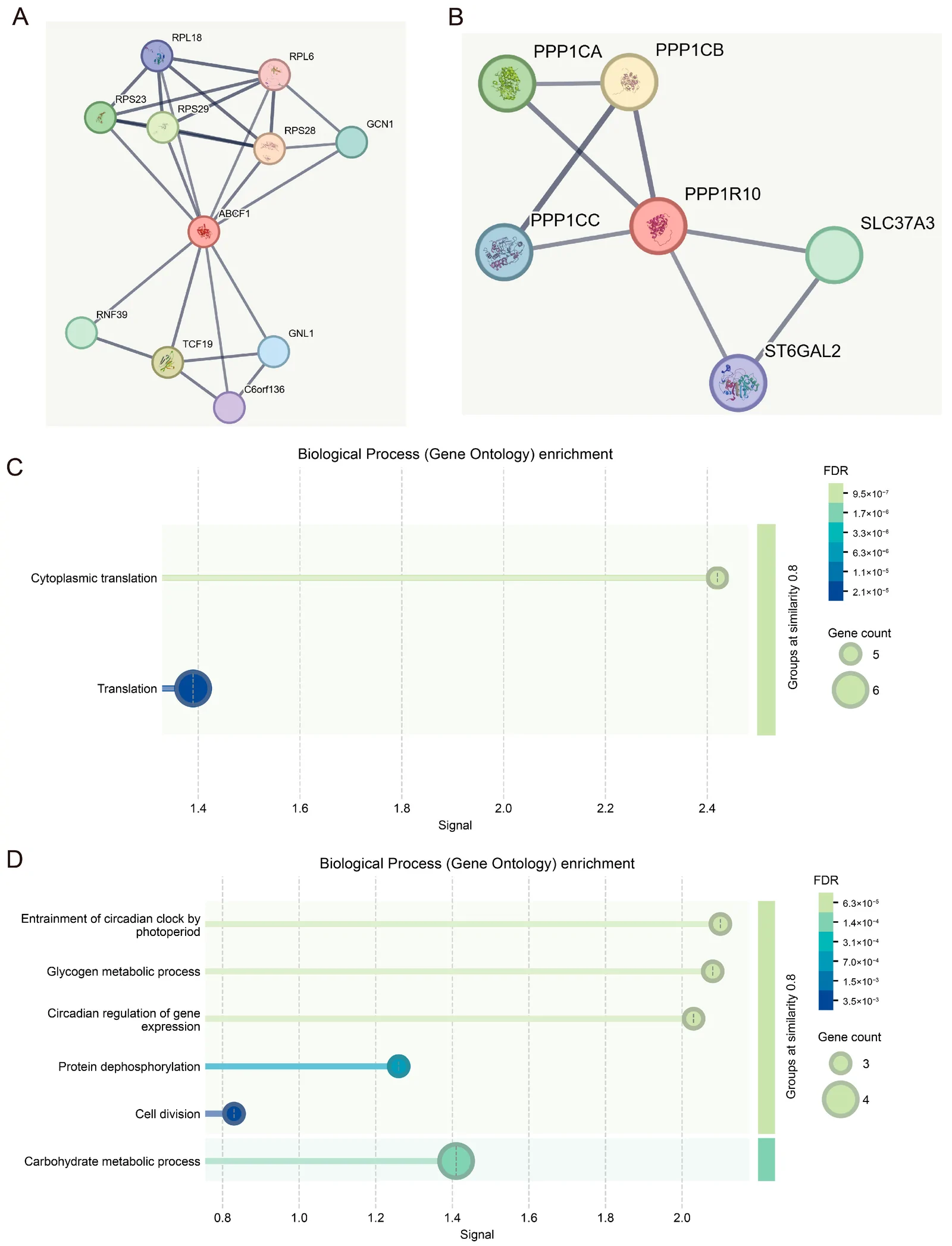
STRING protein–protein interaction networks and GO biological-process enrichment. (**A**) ABCF1 network; (**B**) PPP1R10 network; (**C**) GO biological-process enrichment for ABCF1 interactors; (**D**) GO biological-process enrichment for PPP1R10 interactors. Networks were constructed with STRING v12.0 at a confidence threshold of 0.700. The thickness of connecting lines (edges) indicates the confidence score of protein-protein associations; thicker lines represent higher confidence. In (**C**,**D**), symbol size reflects gene count and color intensity indicates FDR significance.

To place the two proteins within their broader functional context, protein–protein interaction (PPI) networks were constructed using the STRING database (v12.0, confidence threshold 0.700), with concurrent GO enrichment analyses. The ABCF1 network comprised 10 core interactors forming 26 interaction edges, with ribosomal large- and small-subunit proteins as the central nodes (Table 3 and Figure 6A). GO enrichment was highly significant for the polysome term (GO:0005844, cellular component; FDR = 2.02 × 10^−13^) (Table 3) and for cytoplasmic translation (GO:0002181, biological process; FDR = 9.47 × 10^−6^) (Table 3 and Figure 6C). The PPP1R10 network comprised five core interactors forming eight edges, centered on the PP1 catalytic subunits PPP1CA, PPP1CB, and PPP1CC, with additional interactors SLC37A3 and ST6GAL2 (Table 3 and Figure 6B). GO enrichment was significant for the PTW/PP1 phosphatase complex (GO:0072357, cellular component; FDR = 1.37 × 10^−9^) (Table 3) and for the biological-process terms glycogen metabolic process (GO:0005977, FDR = 6.30 × 10^−4^) and protein dephosphorylation (GO:0006470, FDR = 7.40 × 10^−3^) (Table 3 and Figure 6D).

**Figure 7:**
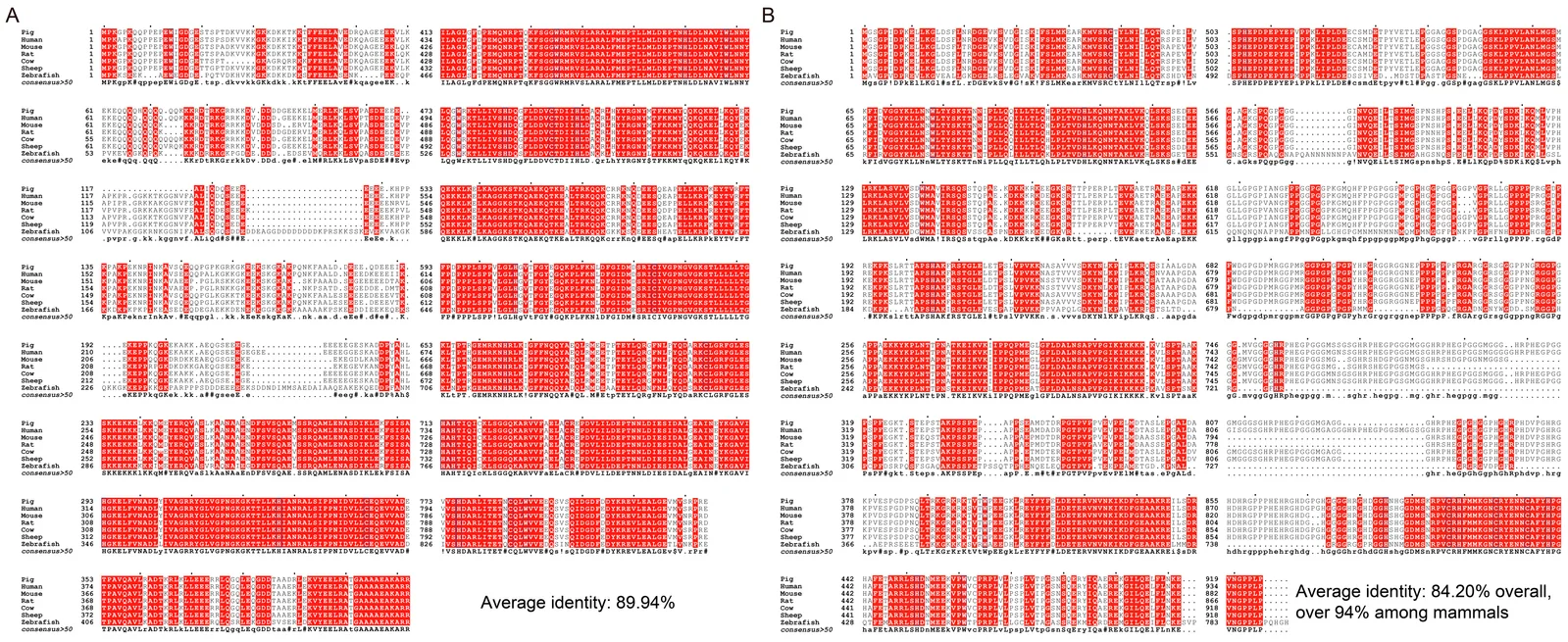
Cross-species amino-acid sequence alignment of porcine ABCF1 (**A**) and PPP1R10 (**B**) across seven species (pig, human, mouse, rat, cattle, sheep, zebrafish). The average sequence identity is shown in each panel. Strictly identical residues are highlighted with a red background and white characters. Columns with strong physicochemical similarity are red and framed in blue. Dots within the species sequences denote alignment gaps, and symbols above the alignment (.) indicate residue conservation.

To assess cross-species sequence conservation, multiple sequence alignments were performed for seven species (pig, human, mouse, rat, cattle, sheep, and zebrafish). ABCF1 exhibited an overall mean sequence identity of 89.94% across all species (Figure 7A), with the zebrafish sequence showing more pronounced divergence. PPP1R10 displayed an overall mean sequence identity of 84.20% (Figure 7B); however, within mammals, sequence identities all exceeded 94%, indicating that the core functional domains are highly conserved in mammals.

## 4. Discussion

Genomic safe harbor (GSH) loci are indispensable for predictable transgene integration in livestock biotechnology, yet only a limited number of porcine safe harbors—including *Rosa26* [44], *H11* [12], *GAPDH* [11], and *ACTB* [10]—have been functionally validated. The *ABCF1*-*PPP1R10* intergenic region on porcine chromosome 7 represents a promising candidate GSH, but the regulatory architecture and functional properties of its flanking genes remained uncharacterized. In the present study, we systematically dissected the 5′-flanking regulatory regions, core promoter activities, protein physicochemical properties, structural conformations, and interaction networks of porcine *ABCF1* and *PPP1R10*. Our integrated bioinformatic and experimental analyses define the minimal functional promoters of both genes, reveal their divergent regulatory logics, and provide a structural–functional framework that supports the safe-harbor potential of this locus. Together, these results directly address our central aim of defining the core promoters and evaluating the safe-harbor potential of this locus.

The contrasting promoter structures of *ABCF1* and *PPP1R10* provide mechanistic insights into their transcriptional regulation. *ABCF1* displays a canonical eukaryotic promoter configuration—TATA, CCAAT, and GC boxes—along with three CpG islands, features commonly associated with broadly expressed genes. In contrast, *PPP1R10* harbors three TATA boxes and lacks CpG islands, suggesting a distinct regulatory logic. In eukaryotic transcription, the TATA box serves as the binding site for TFIID [45,46], and tandem TATA elements may provide redundant binding platforms or modulate transcription start site selection through alternative TFIID positioning [47]. This architectural dichotomy is mirrored in the transcription factor binding landscapes: MAFG::NRF1 and SP1 emerged as the most significant predicted regulators of *ABCF1*, whereas Spi1 and FOXC1 exhibited the most significant predicted binding to *PPP1R10*. The functional significance of these predictions is substantiated by the truncation analyses. For both genes, the shortest fragments tested—P217 (−217 to −1 bp) for *ABCF1* and P211 (−211 to −1 bp) for *PPP1R10*—conferred the highest transcriptional activity in both PFFs and PK-15 cells. Notably, P211 of *PPP1R10* in PK-15 cells even exceeded that of the SV40 positive control on average, indicating that this minimal region concentrates potent constitutive activation signals, potentially reflecting synergistic interactions between the two proximal TATA boxes and adjacent cis-regulatory elements. This raises the possibility that *PPP1R10* is subject to stringent transcriptional control, consistent with the essential role of PNUTS in mitotic progression and chromosome stability [48]. The “proximal activation-distal repression” pattern observed for both promoters is consistent with the regulatory logic of many mammalian housekeeping and growth-related genes. For *ABCF1*, the progressive decline in activity with fragment extension points to the presence of negative regulatory elements in the distal 5′-flanking region. This is particularly intriguing given that *ABCF1* is essential for embryonic development—*Abcf1*^−/−^ mice are embryonic lethal at 3.5 days post coitum [49] and its expression is tightly coupled to proliferating and differentiating cell types. The distal negative elements may serve to prevent spurious overexpression that could disrupt translation homeostasis. This regulatory arrangement is consistent with the observation that porcine *ABCF1* harbors functional polymorphisms in its 5′ untranslated region that associate with growth traits such as backfat thickness and teat number [50], implying that the regulatory region we have defined is subject to selective pressure in commercial pig populations. The MAX::MYC-centered regulatory network is particularly relevant: MAX functions as an obligate partner of MYC in driving ribosomal biogenesis and translation-related gene expression, and the co-occurrence of MAX::MYC and NFIC binding sites in the distal *ABCF1* promoter region (approximately 2.9 kb upstream of the ATG) suggests a feed-forward mechanism that couples *ABCF1* transcription to the translational demands of proliferating cells.

For *PPP1R10*, KLF4 was also among the most significant predicted regulators, although its predicted binding site lies approximately 2.7 kb upstream, outside all tested truncation fragments; KLF4 is a multifunctional transcription factor that regulates basal expression of target genes through direct promoter binding and recruitment of coactivators such as p300 [51]. The activity of *PPP1R10* fragments also showed cell-type-dependent patterns: P211 consistently exhibited the highest activity in both cell lines, whereas P1939 showed a more pronounced activity increase in PK-15 cells, hinting at context-dependent regulation. This is consistent with the known involvement of PNUTS in diverse processes including transcription elongation, chromatin remodeling, DNA repair [52], and maintenance of chromosomal stability [48].

The structural analyses of ABCF1 and PPP1R10 reveal a fundamental division of biochemical labor (Table 4). Porcine ABCF1 is an acidic, α-helix-dominant protein (52.67%) containing a P-loop NTPase domain and an ABC transporter signature module. The crystal structure of ATP-bound human ABCF1 has been resolved at 2.3 Å, revealing a unique pre-activation intermediate conformation in which ATP binding stabilizes the active site geometry [43]. This structural rigidity is functionally essential for ABCF1’s role in start codon selection during translation initiation. The intrinsically disordered regions we identified (residues 29–39, 209–221, and 538–581) likely mediate the multivalent interactions required for ribosome association and, as shown for the human ortholog, for transcriptional coactivation through a low-complexity domain. Concordantly, ABCF1’s high-confidence interactions with ribosomal proteins (RPL6, RPL18, RPS23, RPS28, RPS29) and enrichment in cytoplasmic translation pathways align with its established role as a non-transporter ABC protein that regulates translation initiation through eIF2 binding [18,53]. However, the emerging evidence positions ABCF1 as a multifunctional node that extends well beyond translation. Recent reviews have documented its involvement in innate immune sensing, where ABCF1 associates with cytosolic DNA to modulate inflammatory cytokine production, and its low-complexity domain mediates phase separation and transcriptional regulation in embryonic stem cells [19].

In contrast, PPP1R10 is a basic protein (pI 9.25) overwhelmingly composed of random coils (69.41%), with a CCCH-type zinc finger, a TFIIS N-terminal domain (TND), and a PP1 regulatory subunit domain. This conformational flexibility is functionally appropriate for a scaffolding subunit that assembles the PTW/PP1 phosphatase complex, which also includes TOX4 and WDR82. Our PPI network confirmed high-confidence interactions with all three PP1 catalytic isoforms (PPP1CA, PPP1CB, PPP1CC), aligning with the canonical role of PNUTS/PPP1R10 as a nuclear targeting subunit for PP1 [21]. The TND, structurally conserved from TFIIS, is predicted to mediate interactions with transcriptional regulators including TOX4 [54], while the zinc finger domain likely contributes to nucleic acid binding. Beyond this classical function, the PTW/PP1 complex regulates RNA polymerase II promoter-proximal pause release by dephosphorylating CDK9 [55], and other elongation factors, positioning PPP1R10 at the interface of transcription and chromatin regulation. The enrichment of PPP1R10 interactors in protein dephosphorylation and glycogen metabolic process pathways further underscores its role in phosphatase-mediated signaling. Notably, in Drosophila, selective disruption of PNUTS-Tox4 and PNUTS-PP1 interactions impairs chromosomal dispersal and gene expression during oogenesis [54], underscoring the essential role of this phosphatase complex in developmental transitions [56].

Cross-species conservation analyses revealed that the proteins encoded by both genes maintain high sequence identity among mammals (ABCF1: 89.94% overall; PPP1R10: >94% within mammals), indicating that their core functional domains and regulatory mechanisms are evolutionarily conserved. From a translational perspective, the *ABCF1*-*PPP1R10* locus satisfies the fundamental sequence-level criteria for GSH designation: absence of canonical oncogenes within the ±100 kb flanking window, stable basal transcriptional activity, and conserved regulatory architecture. The dual-promoter organization—with divergent core promoters residing within 217 bp and 211 bp of the respective translation start sites—may offer practical advantages for bidirectional transgene expression strategies. However, direct comparison with established porcine safe harbors is informative. The *COL1A1* and *Rosa26* loci have been shown to support higher EGFP expression with lower DNA methylation of the transgene promoter compared to *H11* and *AAVS1* [44], and the *H11* locus enables efficient CRISPR/Cas9-mediated knock-in [12]. Whether the *ABCF1*-*PPP1R10* locus achieves comparable integration efficiency and expression stability remains to be determined through direct experimental comparison.

Several limitations of this study warrant acknowledgment. First, the promoter activity assays were conducted in PFFs and PK-15 cells, which may not fully recapitulate the transcriptional landscape across porcine tissues and developmental stages. Both *ABCF1* and *PPP1R10* exhibit ubiquitous expression in pig tissues, but cell-type-specific regulatory mechanisms cannot be excluded. Second, the transcription factor binding predictions require experimental validation through chromatin immunoprecipitation and site-directed mutagenesis of the predicted MAFG::NRF1, SP1, Spi1, and FOXC1 binding sites. Third, structural predictions for both proteins should be interpreted with caution. The ABCF1 SWISS-MODEL (template 5zxd.2.A, 99.26% identity) has a moderate GMQE (0.51) because the template covers only the core region of the full-length sequence, while the modelled core itself is locally reliable (QMEANDisCo = 0.79 ± 0.05). The PPP1R10 SWISS-MODEL was built on the AlphaFold DB template AF-Q767K9-F1 (100% identity, full-length coverage; GMQE = 0.57), which reflects the low average pLDDT of the template itself (57.81); no mechanistic inference is drawn from the low-confidence regions. The conformational dynamics of their disordered regions merit further investigation. Future studies should include ATAC-seq and ChIP-seq profiling across porcine tissues, functional validation of the core TF regulatory axes, and CRISPR/Cas9-mediated knock-in of reporter cassettes to directly assess the integration efficiency and long-term expression stability of this locus in transgenic pigs. In addition, the serially truncated fragments tested may not capture all distal regulatory elements, and the cross-species promoter comparison remains computational until experimentally validated.

## 5. Conclusions

In summary, this study provides the first systematic characterization of the porcine convergent *ABCF1*-*PPP1R10* gene pair. We delineated the minimal functional promoters P217 and P211, which exhibited the highest transcriptional activity in PFF and PK-15 cells. We further revealed divergent promoter architectures, complementary protein structural and functional properties tailored to translational regulation and phosphatase signaling, respectively, and an oncogene-free ± 100-kb flanking region. Collectively, these findings provide fundamental evidence supporting this interval as a promising novel porcine genomic safe-harbor candidate, while also contributing to the broader understanding of gene regulation in livestock; nevertheless, site-specific knock-in and in vivo validation remain necessary to confirm its biosafety and long-term transgene expression stability. Future work should evaluate the integration efficiency, transgenerational expression stability, and genome-wide off-target effects of this candidate locus in transgenic pigs.

## Figures and Tables

**Table 1:** Prediction of core promoter sequences in the 5′-flanking regions of porcine ABCF1 and PPP1R10. All coordinates in this table are relative to the start codon ATG of the porcine *ABCF1*-*PPP1R10* convergent gene pair (ATG = +1; upstream positions are negative), matching the ATG-relative x-axis of Figure 1. These coordinates refer to the NNPP-predicted core promoter candidates and are independent of the boundaries of the serially truncated fragments tested in the dual-luciferase assays.

Gene	No.	Start (bp)	End (bp)	Score	Promoter Sequences
*ABCF1*	1	−2176	−2126	0.99	CCCCATGGCATAAAAAAGGCTGAGGATCAGATCTGAGCTGCTGTTGCAAC
	2	−2007	−1957	0.99	CATGACTTTAAATAAATACCCACCATGGGAGTTCCCGTTGTGGGCTCAGT
	3	−1734	−1684	0.95	AGACATAATAAATAAATACCCACCATAACCGATGAAGATCCTAATGTCTC
	4	−767	−717	0.93	GGTAGTAGTTAAAAAAGTGAACCCAAGCTTCACTGCACTGTGATTTCTGT
	5	−53	−3	0.95	CGGAAGCGGAAATAACGCCCGGCGCCGCCAGAGTTGCCGTAACTGCCACC
*PPP1R10*	1	−2329	−2279	0.86	ATTTCCAGAATATAAATACTTGGGAAAATATAGCAGAAAGTGAATATTTA
	2	−2217	−2167	0.99	AATGGGTATTAAAAAGTTGTCCCTGGGTCCCATTCCCAGCAGAAGACACT
	3	−1333	−1283	0.87	AACATGATCTTATAAGAAGCCTGTTTTCTTTTCTGCAGGAAATATAGTCG

**Table 2:** Key physicochemical parameters of porcine ABCF1 (K7GL02) and PPP1R10 (Q767K9).

Parameter Category	ABCF1 (K7GL02)	PPP1R10 (Q767K9)
Basic sequence features	824 aa, acidic protein	925 aa, basic protein
Theoretical isoelectric point (pI)	5.94	9.25
Molecular weight	93.92 kDa	97.60 kDa
Charge bias	11 more negatively charged residues	17 more positively charged residues
Most abundant amino acids	Glutamate (12.5%), Lysine (11.4%)	Glycine (16.2%), Proline (14.3%)
Instability index	49.29 (unstable)	54.41 (unstable)
Grand average of hydropathicity (GRAVY)	−0.985 (highly hydrophilic)	−0.844 (highly hydrophilic)

**Table 3:** Core interactors and significantly enriched pathways of porcine ABCF1 and PPP1R10.

Protein	Core Interactors	Interaction Coefficient Range	Protein Function	Significantly Enriched GO Terms (FDR)
ABCF1	RPL6, RPL18	0.787–0.794	Ribosomal large subunit assembly	Polysome (CC; 2.02 × 10^−13^)
	RPS23, RPS28, RPS29	0.858–0.888	Regulation of cytoplasmic translation	Cytoplasmic translation (BP; 9.47 × 10^−6^)
	GCN1, GNL1, RNF39, TCF19, C6orf136 (C7H6orf136)	0.787–0.881	Translational regulation; MHC-region-encoded proteins	—
PPP1R10	PPP1CA, PPP1CB, PPP1CC	0.750–0.880	PP1 phosphatase catalytic core	PTW/PP1 phosphatase complex (CC; 1.37 × 10^−9^); Protein dephosphorylation (BP; 7.40 × 10^−3^)
	SLC37A3	0.768	Sugar phosphate exchanger	Glycogen metabolic process (BP; 6.30 × 10^−4^)
	ST6GAL2	0.72	Sialyltransferase (protein glycosylation)	—

**Table 4:** Integrated comparison of porcine ABCF1 and PPP1R10 protein functional profiles.

Comparison Dimension	ABCF1 (K7GL02)	PPP1R10 (Q767K9)
Biochemical property	Acidic protein, rigid conformation	Basic protein, highly flexible conformation
Core conserved domains	P-loop NTPase, ABC transporter signature	CCCH zinc finger, TFIIS, PP1 regulatory domain
Dominant secondary structure	α-helix (52.67%)	Random coil (69.41%)
Core interaction targets	Ribosomal subunits (RPL/RPS)	PP1 catalytic subunits (PPP1CA/CB/CC)
Core functional enrichment	Cytoplasmic translation, polyribosome assembly	PTW/PP1 phosphatase complex, glycogen metabolism
Subcellular localization	Cytoplasm + nucleus (nuclear membrane, nucleoplasm)	Nucleus (chromatin-bound)
Sequence conservation	Overall 89.94% identity across species	Overall 84.20%; mammalian > 94%

## Data Availability

The data that support the findings of this study are available from the corresponding author upon reasonable request. The public epigenomic datasets analyzed in this study are available in the NCBI Gene Expression Omnibus under accession numbers GSE229520 and GSE143288.

## Supplementary Materials

The following supporting information can be downloaded at: https://www.mdpi.com/article/10.3390/ani16213416/s1, Table S1: Bioinformatics software and databases used in this study; Table S2: Putative transcription factor binding sites in the porcine ABCF1 promoter predicted by FIMO using the JASPAR 2026 CORE motif database (*p* < 1 × 10^−4^); Table S3: Putative transcription factor binding sites in the porcine PPP1R10 promoter predicted by FIMO using the JASPAR 2026 CORE motif database (*p* < 1 × 10^−4^); Table S4: Detailed physicochemical parameters of porcine ABCF1 (K7GL02) and PPP1R10 (Q767K9); Figure S1: Genomic context and chromatin profile of the porcine ABCF1-PPP1R10 convergent gene pair on chromosome 7 (Sscrofa11.1); Figure S2: Cross-species multiple-sequence alignment of the 5′-flanking promoter regions; Figure S3: Predicted aligned error (PAE) heatmap for the porcine PPP1R10 protein structure (AlphaFold DB model AF-Q767K9-F1).
